# The directed assembly grand challenge network

**DOI:** 10.1186/s13065-017-0303-3

**Published:** 2017-08-01

**Authors:** J. A. R. Rose, P. R. Raithby, C. Makatsoris

**Affiliations:** 1 School of Aerospace, Transport and Manufacturing (SATM), Cranfield University, Cranfield, MK43 0AL UK; 2 Department of Chemistry, University of Bath, Bath, BA2 7AY UK

## The grand challenge

In the modern world the design, development and use of new materials is having a major economic and societal impact in areas as diverse as: healthcare, energy, food production, transport, construction and electronics. The grand challenge for the coming decades is to be able to control the properties and functions of these new materials through intelligent design in environmentally friendly, energy efficient ways that are not possible presently. The ultimate goal is to be able to design and fabricate a material with a targeted range of properties with 100% success.

All the current and future materials, with dimensions from the nanoscale to the macroscopic scale, are and will be, built through the assembly of individual atoms or small molecular building blocks. Through our determination to meet the grand challenge by controlling the assembly of these building blocks and developing the understanding of the science as we move up though the length scales, we will continue to make major breakthroughs in both science and engineering along the way that will transform the world as we know it.

The directed assembly network (DAN) was one of three grand challenge networks originally funded by the EPSRC Chemistry Programme back in in 2010, and has been going from strength to strength since that time. The original vision of the DAN was to develop methodologies that will afford exquisite control over the preparation, properties and function of materials that can be assembled into complex pre-designed structures. Since then, without losing the original vision, it has expanded to include understanding how materials may be disassembled as well as assembled, and the ideas are not restricted to the solid state but solution and gas phase processes are also being included. There is also more focus on the scale-up of the new materials so that they are available in industrially useful quantities.

Under the umbrella of this grand challenge, chemists, physicists, biologists, mathematicians, and chemical, mechanical and electronic engineers are combining in wide ranging collaborative projects to develop new materials, with targeted properties and functions that will ultimately meet some of the following current world challenges:

- The development of new medicines and therapies that can cure currently unbeatable diseases [1].
- The development of unlimited and inexpensive fuel sources through new ways of transporting, storing and generating energy.
- The development of new materials and measurement devices will enable the rapid and reliable detection of concealed explosives, weapons and chemicals for example, and can help to protect against crime and terrorism.
- The development of efficient methods for the purification and reclamation of water.
- The development of new agrochemicals and agricultural processes that will safely enhance food production and provide world food security.

It should be emphasised that these are 50 year goals that require the fundamental science and engineering to be understood and developed in ways that are not currently possible [2]. Essential to this vision is a multidisciplinary approach involving the collaboration of the full range of physical and life scientists together with engineers, mathematicians, and economists. Buy in from industry, end users, policy makers and the general public is also essential if these goals are to be met and the new methodologies and processes adopted.

**Fig. 1:**
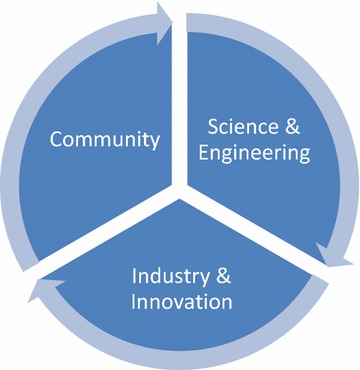
Network focus points

Within DAN a multifaceted approach (Fig. 1) has been developed and employed towards both setting and meeting the many targets, in addition to overcoming the many challenges on the way to the absolute goal of being able to prepare and fabricate any material with predesigned properties or functions, whether they are physical, chemical or biological.

## A roadmap to innovation

The roadmap to innovation [3] was constructed by the community-represented by DAN through years of consultations, fostering debate and continuous engagement via: themed meetings, sandpits, network awards (proof of concept projects) and strategic goals [3]. The network looked 5–50 years into the future, capturing short, medium and long term goals.

The UK position and world challenges are set out in the roadmap to highlight the importance that new materials provide when it comes to solving the grand challenges of this century.

**Fig. 2:**
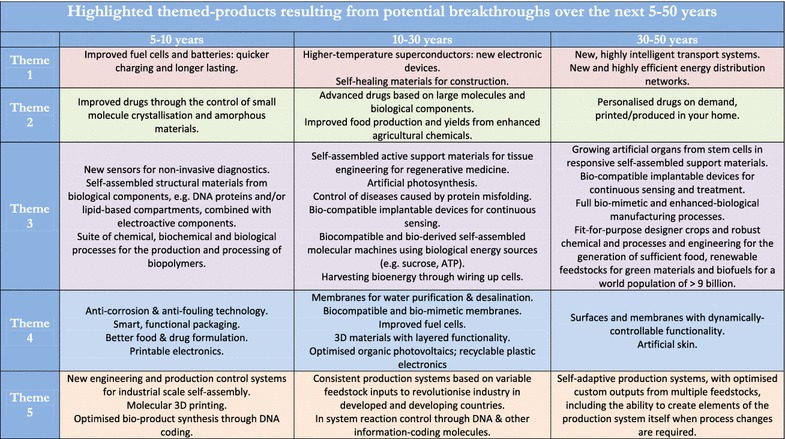
Highlighted themed-products resulting from potential breakthroughs over the next 5–50 years

Challenges include, in addition to those already mentioned:

- The development of medicine and implants tailored to each individual;
- The creation of green alternatives to scarce resources;
- Capturing pollutants;
- Enhancing crop production;

More generally, the development of exciting innovations, such as smart materials, new catalysts, organic computing and high-temperature superconductors, will lead to disruptive technologies that will transform society. Some products are highlighted according to theme (see the section below for information about network themes), in Fig. 2, which will result from potential breakthroughs over the next 5–50 years. See our roadmap for more in depth information [3]. For example, over the next 5–10 years improved fuel cells will lead to quicker charging and longer lasting batteries. In 10–30 years there will be self-assembled active support materials for tissue engineering and regenerative medicine. Within 30–50 years we envisage that personalised drugs and medicines will be available on demand, locally, within your home.

## Network themes and challenge streams

The science is set out across five overlapping themes in the roadmap [3]:

1. Functional hybrid materials.
2. Nucleation and crystallisation.
3. Biological and biomimetic systems.
4. Surfaces and interfaces.
5. Intelligent chemical reactors.

**Fig. 3:**
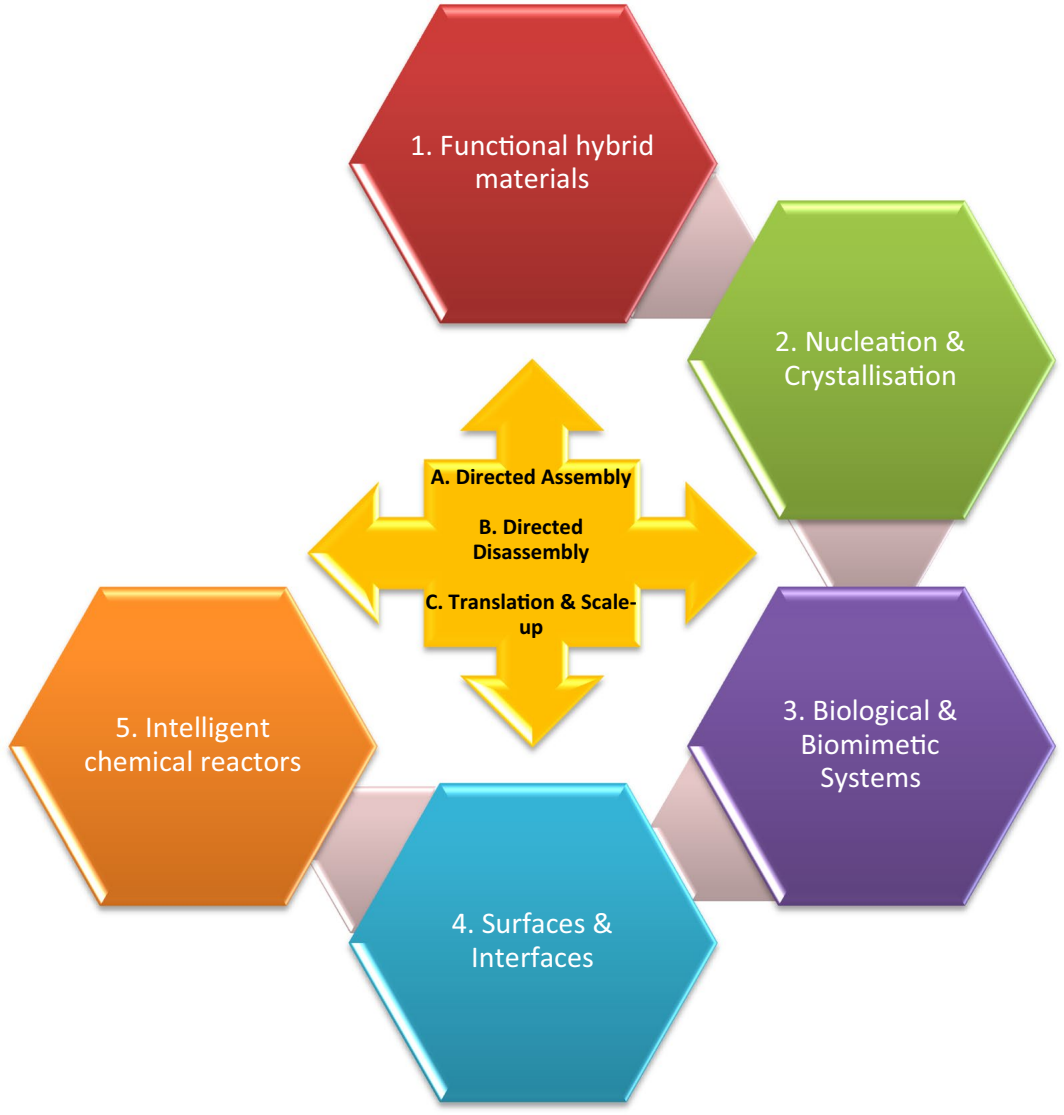
Network themes and challenge streams

The themes encompass the three challenge streams (Fig. 3):

A. Directed assembly.
B. Directed disassembly.
C. Translation and scale-up of the research towards manufacturing.

The first challenge stream, directed assembly, is led by Professor Paul Raithby. This stream will continue to extend the existing findings and roadmap of the network, on the assembly of molecular building blocks into pre-designed structures in condensed and liquid phases. The range of methodologies that are available or are currently being researched together with future research directions for how such assembly processes work, will be investigated, catalogued and recorded by the network. This stream covers the range of molecular building blocks, from inorganic to biological systems, including materials from perovskites to polymers, for studies in the solid-state, in solution or on surfaces or at interfaces.

The second challenge stream, directed disassembly, is led by Professor Chick Wilson. There are numerous applications where the condensed material must be dispersed or dissolved to release its components to deliver the desired functionality. This stream is a new key feature of the evolved network for 2017 and beyond. Here we explore how to direct the disassembly of these materials into their constituent building blocks, releasing activity or action on appropriate triggering. As for directed assembly, we will record, catalogue and disseminate the methods and future directions that can enable the controlled disassembly of suitably designed condensed materials for the range of building blocks considered above.

The third challenge stream, translation and scale-up, is led by Professor Charalampos (Harris) Makatsoris and is a crucial new feature for phase three of the network that reflects the broadening of the network focus to include the translation of discoveries into applications. The roadmap [3] highlights certain short term capabilities and developments that are now moving rapidly towards the application phase, entering the higher technology readiness levels (TRL) 3–4 and application domains. Although the network focus will remain in TRLs 1–3, with this stream, chemists, engineers and practitioners will be brought together to explore and define the pathways for the translation of those scientific discoveries to potential future application domains.

Prospective manufacturing routes for those materials will also be explored. However, this stream will not be limited to such unidirectional exchange but rather will embrace bidirectional interaction with end-users allowing the development of multi-disciplinary collaborations to define applications or address particular engineering and industrial challenges.

This stream has two focus areas:

1. Formulation, which entails the ability to control microstructures of multi component materials including complex fluid formulations, guest/host systems, polymeric materials etc., to deliver the range of functions that targeted applications demand.
2. Scale-up, which involves the understanding and the control that is required for manufacturing those materials at market scales. Ultimately, data-driven, mathematical techniques and design tools would be needed for manufacturing process design.

## The directed assembly network

The network has grown to over 1000 members, since its inception in 2010, and hosts several highly acclaimed meetings each year. Early career researchers (ECRs) are at the heart of the network and benefit greatly from access to, and networking with, our world-leading members and senior management team. The network is now embedded into the culture of the UK landscape and continues to work at the leading edge.

The network has proven very successful in fostering this research, helping to maintain or propel the UK to the forefront of the research areas. The network has awarded over £325,000 in pump-priming, travel and seed-corn grants [4]. Over 80 new collaborations have been formed and £50 Million of subsequent grants are linked to and/or supported by the directed assembly network [5].

ECRs are a key focus and as such, the network has just over 25% ECR membership. Specific meetings are held to promote ECRs, along with a network mentoring system. We have now seen many of the ECRs develop and achieve more senior positions and some have now joined the Network Management Team.

The range of materials and methods encompassed by the themes, in conjunction with the challenge streams, further help to place the network in a position to cover a wide variety of potential future applications that will support the four key targets that underpin UK prosperity: productivity, connectedness, resilience and health [6]. Those include: drug delivery, sensors, photovoltaics, smart coatings, self-healing components and surfaces to name a few.

The network provides opportunities to establish multidisciplinary collaborations to address large, application-driven problems. For example, determining the optimal formulation and translation and manufacturing pathways for guest/host systems that can act as smart responsive coatings or sensing surfaces.

Advances in some of the short term (2–5 year) research goals set out in the roadmap are highlighted by the inclusion of network-funded proof of concept project examples within the themed sections [3].

## Directed assembly themed series

Within this invited directed assembly themed series, the community’s progress towards tackling the grand challenge is showcased by the invited papers and reviews presented by network members. These are accompanied by published meeting reports which record and document the latest directed assembly and disassembly themed activities.
